# Supplementary figures and images for: Inkjet-printed transparent electrodes: Design, characterization, and initial in vivo evaluation for brain stimulation
Source: PLoS One. 2025 Apr 1;20(4):e0320376. doi: 10.1371/journal.pone.0320376 (PMC11960977; doi:10.1371/journal.pone.0320376)

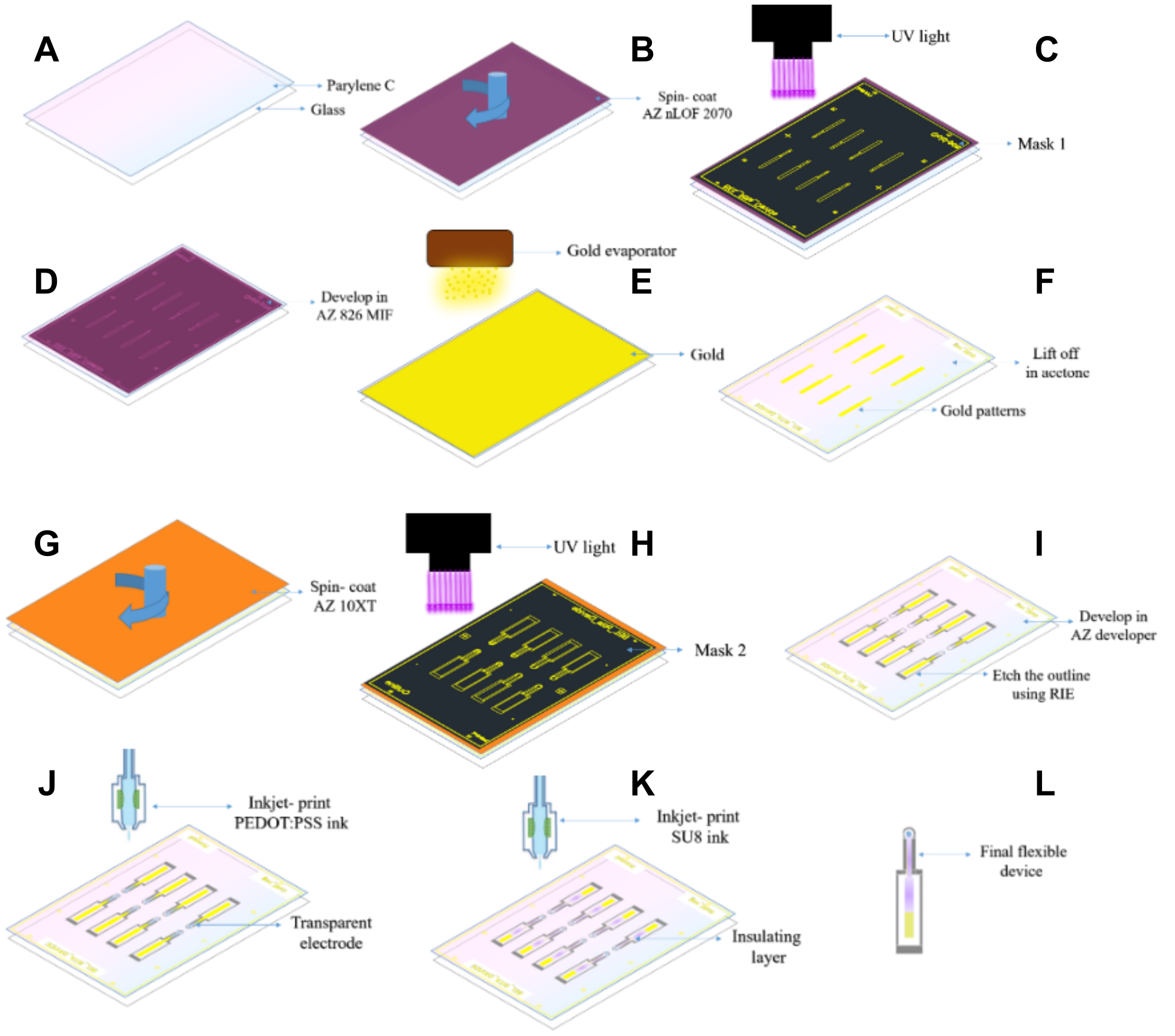

Supplement: S1 Fig — A: PaC deposition. B: Spin-coating AZnLOF 2070. C: UV Exposure. D: Developing. E: Gold evaporation. F: Lift-off. G: Spin-coating AZ 10XT. H: UV Exposure. I: Developing and reactive ion etching. J: Inkjet-printing PEDOT:PSS. K: Inkjet-printing SU-8. L: Final device. (TIF) [file pone.0320376.s001.tif]

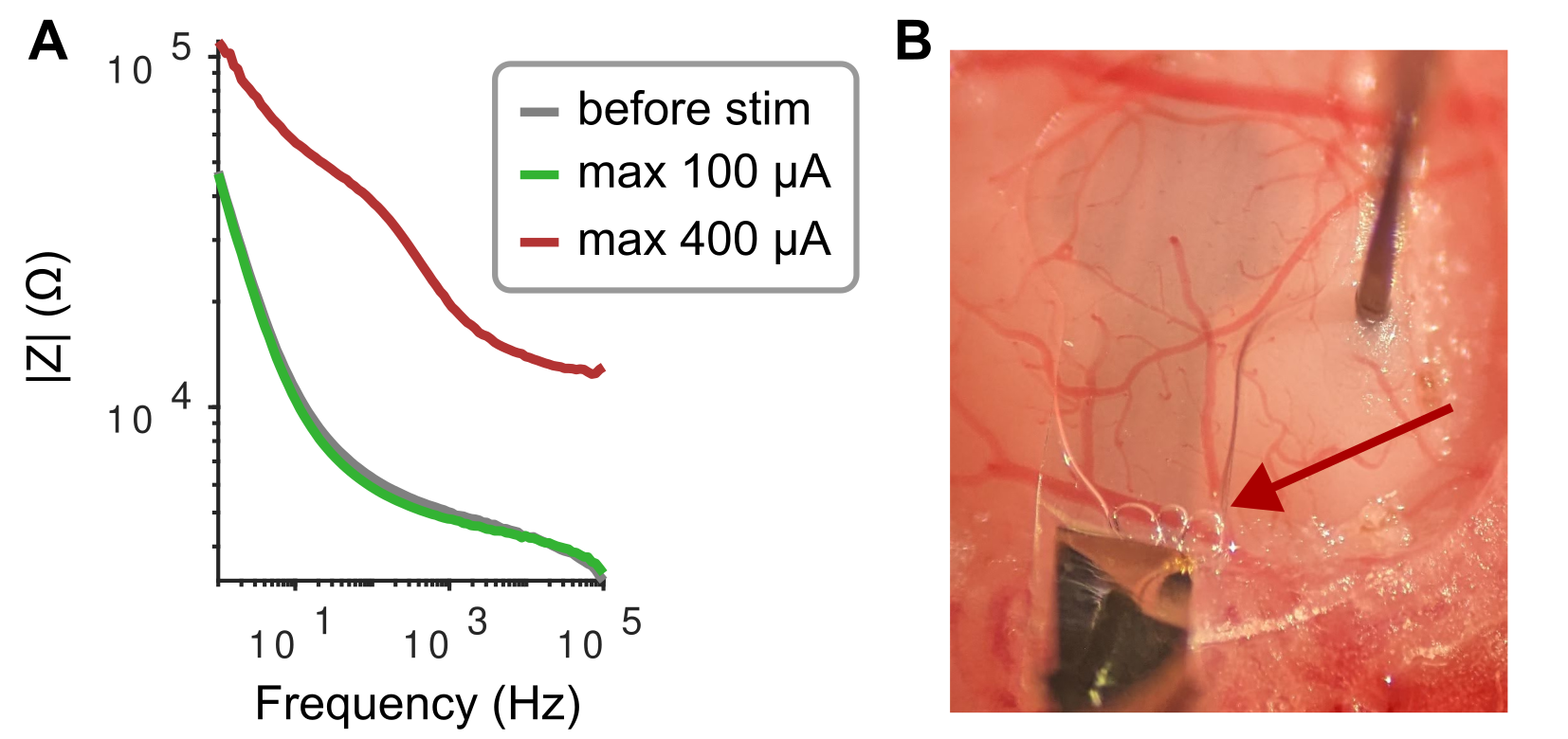

Supplement: S2 Fig — A: In vivo electrical impedance spectroscopy measurements before applying the stimulation (gray) after applying a set of stimulation with an amplitude of maximum 100 μA (green) and after a subsequent stimulation set with an amplitude of maximum 400 μA (red). The comparable impedance values following stimulation at 100 µ A indicate that the electrode performance is unchanged and also suggest the potential for electrode reusability in other experiments. However, higher current intensities may cause irreversible damage, degrading electrode performance. B: Occurrence of bubbles (indicated by the red arrow) after the second set of stimulations. Note that they appeared at the intersection between PEDOT:PSS and the gold connector. (TIF) [file pone.0320376.s002.tif]
