# Supplementary material for: Inkjet-printed transparent electrodes: Design, characterization, and initial in vivo evaluation for brain stimulation
Source: PLoS One. 2025 Apr 1;20(4):e0320376. doi: 10.1371/journal.pone.0320376 (PMC11960977; doi:10.1371/journal.pone.0320376)
Supplement: Attachment — Submitted filename: ReviewReport_PONE-D-24-40579.pdf [file pone.0320376.s003.pdf]

Review of
"Inkjet-printed transparent electrodes for electrical brain stimulation (PONE-D-24-40579)"

The authors report the development and preliminary testing of an electrode for neurostimulation. A hybrid cleanroom dependent and clean room free inkjet printing approach has been used for electrode fabrication. While similar approaches have been used in the past to develop electrodes for neuroscience applications, the manuscript discusses results in a mouse model, which advocates for consideration of this manuscript. The experimentation for use case in animals is limited but the work covers fabrication, and the analysis is quite detail. I particularly commend the discussion of how the 300 V/m electric field compares to the common brain stimulation applications.

I suggest the following to be implemented in the revised manuscript before it can be considered further:

- It should be categorically established how this work compares with existing inkjet printed PEDOT:PSS electrodes for implantable applications. One important reference is https://doi.org/10.1002/pssa.202100683 (Transparent, Low-Impedance Inkjet-Printed PEDOT:PSS Microelectrodes for Multimodal Neuroscience). A benchmarking/comparison table is necessary to bring out novelty/USP of the current work. Also, sensing and transduction should not be the only comparative factor as it's quite intuitive that in neural and cardiac systems, the dual use capability of electrodes is obvious.
- It is my opinion that the title needs revisiting. There are only primitive studies for deployment in animal models and thus the current form of title is an ambitious representation of the work "Inkjet-printed transparent electrodes for electrical brain stimulation". I believe making the title modest is necessary to indicate the true nature of the work, something like "Inkjet-printed transparent electrodes: Towards electrical brain stimulation", or "Promising Approach…", etc.
- Limitations of the work have not sufficiently been highlighted.
- The abstract is very superficial in it's current form with electric field value from simulation highlighted. More quantified data should form part of the abstract.
- Page 5, Line 186: "PEDOT:PSS vs Ag/AgCl ([-0.6 0.8] V)" – I believe "[-0.6 0.8] V" is being used to discuss the potential window. I am of the opinion it should be replaced with explanatory representation to improve paper readability.
