# Supplementary material for: Inkjet-printed transparent electrodes: Design, characterization, and initial in vivo evaluation for brain stimulation
Source: PLoS One. 2025 Apr 1;20(4):e0320376. doi: 10.1371/journal.pone.0320376 (PMC11960977; doi:10.1371/journal.pone.0320376)
Supplement: Attachment — Submitted filename: Response to Reviewers.docx [file pone.0320376.s005.docx]

We thank the Editor and the Reviewers for their comments and suggestions. We addressed the points one by one below, including the editorial ones that we split to improve clarity. Thanks to the reviewing feedback, we believe that the manuscript is now largely improved.

**Additional Editor Comments:**

The manuscript clearly articulates the objectives, methods, and findings. The technical language is appropriate for a specialized audience, though some sections could benefit from simplification to improve accessibility.

We thank the editor for their positive assessment and helpful comments on our manuscript. We address each comment individually below.

1) The organization is logical and flows well from introduction through to the discussion. Minor improvements in transition sentences between sections could enhance readability.

In response to the editor's suggestion to improve flow, we have added transition sentences throughout the manuscript.

We added the following sentence at the beginning of the section titled Electrical characterization (line 166): *“We began by measuring the overall thickness of the printed PEDOT:PSS layers, as this variable likely influences both the charge storage capacity of the electrodes (and thus the injectable current) and the device transparency.”*

We added the following sentence at the beginning of the section titled Transmittance measurements (line 234): *“We characterized the transparency of the devices to find a thickness that balances sufficient current injection with high transparency.”*

2) Figures are informative and generally well-organized. Some legends, such as for Figures 2 and 4, could be more descriptive for readers to quickly interpret the data.

We have added sentences to the figure legends to facilitate a clearer and more immediate understanding of the data.

The legend for Figure 2 now reads (new text is underlined):

*Figure 2. Electrical properties of inkjet printed electrodes. A: Thickness of electrodes made of a different number of printed PEDOT:PSS layers (n = 5, 3, 5, 2, 2 electrodes for 1-5 layers respectively). B: Electrical impedance spectroscopy measurements for electrodes made of different numbers of inkjet printed PEDOT:PSS layers. Thick lines represent mean values across electrodes and thin lines the error bars estimated as standard deviation (n = 9 electrodes for 1 printed layer and and n = 3 for the others). Due to their low impedance, the electrodes are compatible with commercially available electrical stimulators. C: Cyclic voltammetry (CV) measurements for electrodes made of different numbers of inkjet printed PEDOT:PSS layers. Larger areas enclosed by the cyclic voltammetry curves indicate higher charge storage capacity of the electrodes. D: Charge storage capacity (cathodic: light gray, total: dark gray) estimated from the cyclic voltammetry measurements (calculated from the areas under the cyclic voltammetry curves) for different numbers of layers. E: Example traces (for one and three layers PEDOT:PSS) from pulse tests to estimate the maximum current that can be applied using the electrodes. Dashed lines represent the water window ([-0.6 0.8] V), defining the voltage range where the electrode/electrolyte interface remains stable and avoids significant water electrolysis. F: Maximum current that can be injected using the electrodes before reaching the water window, beyond which undesirable side reactions may occur.*

The legend of Figure 4 now reads (new text is underlined):

*Figure 4. Combined in vivo experiment and computational modeling to estimate the electric field generated by the inkjet-printed electrode. A: The flexible printed PEDOT:PSS electrode is placed on the mouse brain for stimulation, together with a screw counter electrode in the front to close the circuit. A rigid electrode is used to record voltage fluctuations due to a sinusoidal current stimulation (10 Hz). B: The component of the electric field perpendicular to the brain surface (Ez) is estimated by taking the difference of the amplitude of the recorded sinusoidal voltages at multiple depths (schematic representation). Electric field measurements offer insights into the potential effects of the stimulation at specific spatial locations in the brain. C: Estimated electric field (Ez) for four different currents applied, indicated by different colors. Lines indicate a double exponential fit for each stimulation amplitude. The top plot shows the linearity between current applied and the electric field (both of which are normalized by the values corresponding to the maximum current applied). The black line is the identity line. D: Geometry used for the FEM to simulate the experimental conditions. The stimulating and counter electrodes match the geometry and location of the experiment. E: Representation of the FEM meshing with different colors representing different tissue types (electrode, dura, CSF, brain). Each voxel represents a volume element within the model, where equations governing current flow are solved to estimate the current distribution. F: Experimental data and simulation results for a model in which three parameters (the conductivities of the brain and CSF and the thickness of the CSF layer) were optimized. The model, with optimized parameters, accurately reproduces the experimental data. G: Comparison of the optimal parameters identified with the means and standard deviations derived from multiple studies and reported by Sim4Life (see Materials and Methods). Yellow rectangles represent the minimum and maximum values reported in the literature, which were used as the bounds for the optimization procedure. The CSF thickness varies depending on the location in the brain and therefore we left quite large boundaries for the optimization. The optimized parameters closely match those reported in the literature. H: Predicted electric field beneath and close by the stimulating electrode. Colors indicate electric field magnitude as calculated by the model that best reproduces the experimental data. I: Dependence of the electric field magnitude on the depth directly below the center of the stimulation electrode. The electric field rapidly decays with increasing depth in the brain.*

3) The introduction provides solid context for the need for transparent electrodes. However, adding a brief comparison of this approach to similar recent works could strengthen the motivation for the study.

In response to comments from the editor and Reviewers 1 and 2, we have reorganized and expanded the Introduction to better compare our work with previous studies. Specifically, we have added the following text after the section introducing the need for transparent electrodes (line 83): “*An alternative strategy is to use nanotubes, nanowires or nanomeshes because of the minimal alteration of the light path offered by these structures [38,39]. However, the biocompatibility of the metals often used in these structures has not yet been fully proven. Fabricating devices using transparent materials represents another option. For instance, indium-tin-oxide (ITO) has been used to fabricate transparent devices. However, this inorganic material is expensive, brittle and limited in terms of flexibility [40]. Graphene is an excellent alternative for transparent electrodes [41–43] but cleanroom fabrication remains less cost-effective. Furthermore, the capacity for charge storage and injection with this material may be restricted [43]. Ideally, electrodes for brain stimulation and concurrent imaging should be highly biocompatible, transparent, and capable of injecting sufficient charge to generate electric fields comparable to those used in established human stimulation protocols.”*

We have incorporated the following sentences into the latter part of the Introduction (line 111): *“One example is a recent study [55] that proposed directly inkjet printing PEDOT:PSS electrodes onto the scalp of human subjects (rather than pre-manufacturing them [56]) for electroencephalography (EEG) applications, as this could improve electrical contact, which is often affected by the presence of hair [57]. Thin films of PEDOT:PSS can also be highly transparent [58,59]. Notably, a study [60] demonstrated that inkjet-printed PEDOT:PSS electrodes, using a large volume insulated except for a small electrolyte-contact area, can achieve transparency and ideal electrical properties for localized recording or stimulation. This is attributed to the small contact area combined with the material's volumetric capacitance. This work highlights the feasibility of inkjet printing PEDOT:PSS for fabricating electrodes for brain stimulation.”*

4) Consider briefly explaining terms like “2-photon imaging” and “finite-element modeling” for a broader audience who may not specialize in these methods.

When introducing 2-photon imaging in the Introduction, we have now included the following parenthetical explanation (line 70): *“a microscopy technique capable of imaging deeper into living tissue than other methods”.*

When introducing finite-element modeling in the Introduction, we have now included the following parenthetical explanation (line 125): *“a computational method for simulating physical phenomena by dividing a complex structure into smaller, simpler elements.”*

5) The methods section is comprehensive but could be condensed by removing redundancy in protocol descriptions (e.g., the procedure for inkjet printing PEDOT). Consider adding references for well-established protocols rather than describing them in full detail.

We appreciate the reviewer's feedback regarding the length of the Methods section. We aimed for a comprehensive description of the electrode fabrication process, particularly the inkjet printing of PEDOT:PSS, to ensure reproducibility. Maintaining a detailed protocol is common practice in the field of neural interface fabrication, especially when describing relatively new materials or fabrication techniques, such as those employed in our work. We have carefully scrutinized the Methods section, and we believe that every detail included is crucial for other researchers to accurately replicate our electrode fabrication process. Removing any of these details, in our assessment, would unacceptably hinder the reproducibility of our work. However, to provide further context and background information without adding to the length of the Methods section, we have added a reference to a previous publication that focuses specifically on the ink formulation (line 555): *“Further details on the ink formulation rationale and the mechanical and electrical properties of the PEDOT:PSS films are provided in [54].”* We hope that this addition, while not reducing the length, helps to address the reviewer’s concern by providing a resource for readers seeking more in-depth information on that particular aspect. We are, of course, open to further suggestions from the editor on this matter.

6) The manuscript briefly mentions ethical approval for animal studies, which is good. You could add a sentence on efforts to minimize animal suffering and ensure welfare.

We modified the following sentence in the Methods section (line 624): *“All animal experimental procedures, aimed at minimizing any animal suffering during all surgical procedures, were approved and performed in accordance with the French Ministry of Higher Education, Research and Innovation (approval reference number APAFIS#22182–2019091818381938).”*

7) The results are generally well-presented, but adding statistical markers directly within Figures (like error bars or p-values) could help reinforce findings.

Error bars are shown for all data points where more than one sample was available. Statistical analyses, where necessary, are reported directly in the main text.

8) In the section describing Electrochemical Impedance Spectroscopy, consider expanding on how the impedance values achieved relate to similar electrode materials in other studies.

The specific impedance of an electrode is influenced by multiple factors, including its material, geometrical properties, and the interface with the electrolyte. Consequently, direct comparisons of impedance values are challenging unless all these factors are identical. However, we can compare the conductivity of the materials. As indicated in the manuscript, our measured values align with those reported in the paper where the ink formulation was initially introduced. Furthermore, we have added an extra reference, Bihar et al. (2017). Based on the sheet resistance and thickness of the PEDOT:PSS printed electrodes reported in their Supplementary Figure 1, we estimate a conductivity of approximately 100 S/cm. This value is similar to our findings, even though the ink formulation differs slightly. We modified the following sentence to include this reference (line 178): *“We performed the measurements using both one layer and two layers. We estimated values of 346 ± 3 Ω/◻ and 174 ± 6 Ω/◻ (“ohms per square”, mean ± SD, n = 7 measurements for one printed layer and n = 6 for two layers), corresponding to conductivity values of 131 ± 1 S/cm and 133 ± 5 S/cm, consistent with values obtained with the same ink formulation [54] as well as with a study reporting approximately 100 S/cm for a similar, though not identical, ink formulation [61].”*.

9) The discussion could benefit from a paragraph comparing the PEDOT electrode’s transparency, flexibility, and charge capacity to ITO or graphene-based electrodes.

In the Discussion section, we have added a paragraph that compares the performance and characteristics of ITO, graphene, and PEDOT:PSS. This comparison also includes an analysis of the known limitations of PEDOT:PSS. The new paragraph reads (line 432): *“While in our study we used PEDOT:PSS, the predominant materials for transparent electrodes are graphene and sputtered metal oxides, such as ITO. ITO exhibits high transparency (~90%) and excellent conductivity (~10^4^ S/cm), with a sheet resistance as low as 36 Ω/◻ [68], but its inherent brittleness limits its flexibility. Conversely, graphene demonstrates over 90% transparency across the ultraviolet to infrared spectrum, coupled with high electrical (10^4^ - 10^5^ S/cm) and thermal conductivity, as well as flexibility and biocompatibility [69]. Nevertheless, its high cost and complex synthesis processes may limit its broader use for electrode fabrication. PEDOT:PSS, in contrast, distinguishes itself by combining transparency with flexibility [70], while offering mixed electronic and ionic conductivity. Furthermore, it benefits from commercial availability and ease of processing and manipulation [48,71]. Despite its significant promise, PEDOT:PSS presents certain limitations, including restricted conductivity, flexibility, and mechanical stability [72]. The material's complex structure, which involves both electronic and ionic transport, makes its properties highly sensitive to processing techniques. Furthermore, the mechanical stability of PEDOT:PSS can be problematic, with potential issues such as cracking, delamination, and re-dispersion in aqueous environment [73]. While incorporating cross-linkers can enhance film stability, this often comes at the cost of reduced electronic conductivity [74].”*

10) The application potential is well-covered, though highlighting potential clinical implications beyond research use could expand the relevance of the work.

We now modified a paragraph in the discussion to highlight potential clinical implications of our work. Specifically, the paragraph now reads (line 513): *“While further testing is required to confirm long-term biocompatibility and functionality, our device represents a significant advancement due to its cost-effectiveness and rapid testability. This makes it valuable not only for research but also for clinical applications requiring effective stimulation or recording, as well as optical access or imperceptibility [111]. Transparent PEDOT:PSS electrodes, for instance, are also used in biosensors and wearable health monitoring devices, offering conductivity without sacrificing flexibility or transparency [112]. Furthermore, advanced transparent systems like Carbon Layered Electrode Arrays (CLEAR), composed of graphene and Parylene C, have successfully integrated neuro-electrical signal recording with optical coherence tomography for cortical blood vessel monitoring [113]. This underscores the potential clinical relevance of transparent electrode technologies, including those based on PEDOT:PSS, for future applications.”*

11) The limitations of the study could be discussed more explicitly, particularly any known weaknesses of PEDOT in long-term stability.

Following the suggestion of the Editor and Reviewer 2, we have expanded the Discussion section to more thoroughly address the limitations of our work. This includes consolidating previously scattered points and adding new material. The revised section now reads (line 448): *“Our approach presents several limitations. In applications requiring highly localized electric fields, smaller electrodes would be preferable. While our fabrication method has a demonstrated resolution of approximately 30 μm [54], enabling the printing of multiple stimulation electrodes, their individual charge injection capacities would be reduced. Our tests in PBS confirmed that increasing the number of printed PEDOT:PSS layers enhances the maximum injectable charge, but this comes at the cost of decreased electrode transparency. To achieve higher current densities in applications where transparency is critical or when smaller electrodes are necessary, several strategies could be considered. Modifications to the ink formulation [75], including alternative dopants or functionalization strategies [76], may improve charge injection without compromising transparency. Multi-layer coatings of silver nanowires/PEDOT:PSS films represent another promising approach to enhance charge injection while maintaining or even improving transparency [77]. Future studies should also address the challenge of reliable current delivery at elevated levels to mitigate delamination at the gold/PEDOT:PSS interface. Strategies to achieve effective current delivery at elevated levels may include surface treatments to enhance adhesion [78,79] or the incorporation of a buffer layer to reduce stress. Another limitation is the lack of long-term biocompatibility and functionality data for our electrodes. Although a recent study demonstrated that PEDOT:PSS electrodes can deliver low-frequency electric fields without cytotoxicity [80], and another in vivo study showed that PEDOT:PSS coated electrodes could elicit responses in the visual cortex of mice for over a year [50], the longevity of our specific electrode design in vivo remains to be determined. A longitudinal study in mice, for instance periodically measuring electrode impedance with and without current application, would be necessary to assess long-term performance. Furthermore, potential heating of the electrode and tissue during stimulation requires further investigation. Experiments using thermal cameras and varying current levels will be necessary to characterize these thermal effects. In addition, histological analysis will be needed to assess potential tissue damage, including markers of inflammation, cell death, or gliosis [81].”*

12) The conclusion effectively summarizes the significance of the findings. A final sentence emphasizing the next steps or long-term goals for this research could leave a stronger impression.

We modified the last paragraph of the Discussion to include our short and longer term goals of our research. The paragraph now reads (line 523): *“Although direct tests on the effects of stimulation on brain activity are a priority and necessary, our study suggests the feasibility of using inkjet-printed PEDOT:PSS transparent electrodes for both neural stimulation and imaging. These electrodes offer a unique combination of transparency, flexibility, and conductivity, making them attractive for a range of biomedical applications. Future research will focus on enhancing their long-term stability, integrating them into multi-electrode arrays for complex stimulation patterns, and developing fully printable devices with optimized ink formulations. These advancements could significantly impact neuroscience and bioelectronics research, potentially leading to new clinical tools.”*

13) These suggestions are intended to help enhance the manuscript's clarity, strengthen the arguments, and ensure that the presentation of the research is as accessible and impactful as possible.

We thank again the Editor for the comments and suggestions. We believe the manuscript has significantly improved in clarity and impact.

**Review Comments to the Author**

**Reviewer #1**: Overall, this paper introduced a novel transparent electrode for electrical stimulation, with comprehensive property characterizations. The transparent electrode allows brain imaging tools to monitor the brain activity during the stimulation. I personally like the idea. This paper is high-quality and innovative.

We thank the reviewer for their positive comments. We address their concerns below.

Comments:
1. The PEDOT:PSS electrode presented has a small size with only 1 mm diameter. So, the current density injected can be high. This paper should discuss if the electrode surface is heating up or not, and discuss if it will cause any discomforts to the users if used for person.

We agree with the reviewer that the potential for tissue damage due to stimulation is an important consideration. While we did not observe any visible changes in brain tissue after our stimulation tests, suggesting that the current density remained below the threshold for significant tissue alteration, we acknowledge that we did not directly measure temperature changes. To address this limitation, we have added the following sentence to the Discussion, highlighting the need for future studies to investigate potential thermal effects (line 468): *“Furthermore, potential heating of the electrode and tissue during stimulation requires further investigation. Experiments using thermal cameras and varying current levels will be necessary to characterize these thermal effects. In addition, histological analysis will be needed to assess potential tissue damage, including markers of inflammation, cell death, or gliosis [81].”*

2. In terms of the electrode-skin interface, does a skin preparation is required for mouse experiments? For example, shaving the fur on the skin? And if the electrode is used on human head, does the human hair affect the electrode contact quality?

The electrode was placed directly on the mouse dura mater for epidural stimulation. To clarify this, we have added the term “epidural stimulation” in parenthesis to the main text (line 308) and the Methods section (line 636). While our study used a mouse model, we acknowledge that human hair would likely affect electrode contact quality in clinical applications. A recent study demonstrated that one solution is to inkjet-print electrodes directly onto the scalp after scanning the patient's head geometry. This method allows for optimal contact by conforming the wet ink to the scalp's contours before it dries. We have incorporated this concept, along with the reviewer's suggested reference in point 5, into the Introduction with the following sentences (line 111): *“One example is a recent study [55] that proposed directly inkjet printing PEDOT:PSS electrodes onto the scalp of human subjects (rather than pre-manufacturing them [56]) for electroencephalography (EEG) applications, as this could improve electrical contact, which is often affected by the presence of hair [57]”.*

3. Does a conductive gel or electrolyte saline required during the stimulation to improve the electrode impedance?

Our flexible electrodes did not require the use of conductive gels or electrolytes. We have added the following sentence to the Methods section to clarify this point (line 636): *“Neither conductive gel nor any other electrolyte was placed between the electrode and the dura.”*

4. The paper should discuss if the electrode is disposable or can be used repeated.

While we did not quantitatively assess the reusability of the electrodes, our results suggest that reuse is feasible with careful handling during removal. This is supported by two key observations. First, repeated testing in PBS did not affect electrode impedance, indicating their robustness to multiple uses in a wet environment. Second, as shown in Supplementary Figure 2, the electrode impedance remained largely unchanged before and after the in vivo experiment. Therefore, we expect that if the electrodes are removed before the dura mater significantly dries, they could be recovered and reused. To clarify this potential for reusability, we have added the following sentences to the caption of Supplementary Figure 2 (line 1059): “*The comparable impedance values following stimulation at 100 µA indicate that the electrode performance is unchanged and also suggest the potential for electrode reusability in other experiments. However, higher current intensities may cause irreversible damage, degrading electrode performance.”*

5. I am just wondering if this electrode can be used for EEG brain monitoring, because its very small size and good electrical performance, if yes, it might be a good solution to overcome the obstacle of the human hair, where most of the on-skin flexible electrode cannot. (as discussed in this paper: doi: 10.1109/FLEPS51544.2021.9469782).

We agree with the reviewer that smaller electrodes could offer greater flexibility for navigating the challenges posed by hair in human applications. However, reducing electrode size may necessitate smaller connectors, which could present practical challenges. As an alternative, we previously mentioned in our response to point 3 the possibility of directly printing PEDOT:PSS electrodes onto the scalp, as demonstrated in a recent study. This approach, which directly considers the subject's head geometry, allows for optimal electrode contact. We have incorporated this concept, along with the reviewer's suggested reference, into the Introduction (line 111).

**Reviewer #2**: The authors report the development and preliminary testing of a electrode for neurostimulation. A hybrid cleanroom dependent and clean room free inkjet printing approach has been used for electrode fabrication. While similar approaches have been used in the past to develop electrodes for neuroscience applications, the manuscript discusses results in a mouse model, which advocates for consideration of this manuscript. The experimentation for use case in animals is limited but the work covers fabrication, and the analysis is quite detail. I particularly commend the discussion of how the 300 V/m electric field compares to the common brain stimulation applications.

We thank the reviewer for their valuable comments. We address each point individually below.

I suggest the following to be implemented in the revised manuscript before it can be considered further:

• It should be categorically established how this work compares with existing inkjet printed PEDOT:PSS electrodes for implantable applications. One important reference is<https://doi.org/10.1002/pssa.202100683> (Transparent, Low-Impedance Inkjet-Printed PEDOT:PSS Microelectrodes for Multimodal Neuroscience). A benchmarking/comparison table is necessary to bring out novelty/USP of the current work. Also, sensing and transduction should not be the only comparative factor as it’s quite intuitive that in neural and cardiac systems, the dual use capability of electrodes is obvious.

We thank the reviewer for their insightful feedback, particularly for bringing the highly relevant work by Donaldson and Donaldson, and Swisher (2022) to our attention. We have now incorporated this paper into our manuscript, citing it in the Introduction to highlight prior research using thin inkjet-printed PEDOT:PSS electrodes for stimulation. Building upon this previous work, we then detail our novel contributions to the field. Specifically, the relevant section of the Introduction now reads (line 115): “*Notably, a study [60] demonstrated that inkjet-printed PEDOT:PSS electrodes, using a large volume insulated except for a small electrolyte-contact area, can achieve transparency and ideal electrical properties for localized recording or stimulation. This is attributed to the small contact area combined with the material's volumetric capacitance. This work highlights the feasibility of inkjet printing PEDOT:PSS for fabricating electrodes for brain stimulation”.* Then we introduce our work by stating (line 121): *“Here, we confirmed that inkjet-printed PEDOT:PSS electrodes can exhibit high charge injection capabilities while maintaining high transparency and we further characterized the tradeoff between transparency and charge injection capabilities as a function of electrode thickness. In addition, …”*

In the Discussion, we have clarified the similarities and differences between our study and that of Donaldson and Swisher (line 413): *“In our study, we confirmed the results of Donaldson and Swisher [60] by showing that printed PEDOT:PSS can be used to deliver current. Our estimates indicate that the charge storage capacity of our 350 nm printed PEDOT:PSS electrodes is approximately 1 mC/cm^2^ while Donaldson and Swisher reported 6 mC/cm^2^ for their smallest volumes of PEDOT:PSS electrodes (300 μm outer diameter electrodes). Despite having a lower charge storage capacity, our electrodes may offer higher transmittance. Donaldson and Swisher reported transmittance values of 77% at 510 nm and 68% at 900 nm. Our experimental results using a two-photon microscope showed that the theoretical calculation that we used to relate the transmittance measured using a spectrophotometer to that under two-photon conditions is accurate. We can then predict the transmittance of the electrodes proposed by Donaldson and Swisher to be 0.682 * 0.77 * 100 = 35%, so about 10-15% lower than the one we measured using our thinnest electrodes. Thus, comparing our results with theirs highlights the trade-off between charge injection capabilities and transparency, a central theme of this work. This differs from Donaldson and Swisher's focus, which was on characterizing how varying the volume of PEDOT:PSS, while limiting the surface area in contact with the electrolyte, could yield electrodes with suitable electrical properties for recording or stimulation. Building upon their study, we also demonstrate the in vivo applicability of these electrodes and directly measure electric fields in their vicinity. Furthermore, we developed a finite-element model, with parameters fitted to experimental data, to achieve a full characterization of the electric field, including in the region beneath the electrode.”*

Throughout the manuscript, we have clarified the innovation and goals of this study. For example, at the beginning of the Results section, we now state (line 137): *“Our primary goals in this study are to leverage this process to print conductive polymer electrodes suitable for electrical stimulation, to investigate the relationship between their charge injection capacity and transparency and to test them in vivo.”*

• It is my opinion that the title needs revisiting. There are only primitive studies for deployment in animal models and thus the current form of title is an ambitious representation of the work “Inkjet-printed transparent electrodes for electrical brain stimulation”. I believe making the title modest is necessary to indicate the true nature of the work, something like “Inkjet-printed transparent electrodes: Towards electrical brain stimulation”, or “Promising Approach…”, etc.

We agree with the reviewer that the original title could be improved. We have now changed the title to: “*Inkjet-printed transparent electrodes: design, characterization, and initial in vivo evaluation for brain stimulation*” which, we believe, accurately reflects the work conducted and the initial results of using these electrodes for brain stimulation.

• Limitations of the work have not sufficiently been highlighted.

Following the Editor and Reviewer's suggestion, we have expanded the Discussion section to more thoroughly address the limitations of our work. This includes consolidating previously scattered points and adding new material. The revised section now reads (line 448): “*Our approach presents several limitations. In applications requiring highly localized electric fields, smaller electrodes would be preferable. While our fabrication method has a demonstrated resolution of approximately 30 μm [54], enabling the printing of multiple stimulation electrodes, their individual charge injection capacities would be reduced. Our tests in PBS confirmed that increasing the number of printed PEDOT:PSS layers enhances the maximum injectable charge, but this comes at the cost of decreased electrode transparency. To achieve higher current densities in applications where transparency is critical or when smaller electrodes are necessary, several strategies could be considered. Modifications to the ink formulation [75], including alternative dopants or functionalization strategies [76], may improve charge injection without compromising transparency. Multi-layer coatings of silver nanowires/PEDOT:PSS films represent another promising approach to enhance charge injection while maintaining or even improving transparency [77]. Future studies should also address the challenge of reliable current delivery at elevated levels to mitigate delamination at the gold/PEDOT:PSS interface. Strategies to achieve effective current delivery at elevated levels may include surface treatments to enhance adhesion [78,79] or the incorporation of a buffer layer to reduce stress. Another limitation is the lack of long-term biocompatibility and functionality data for our electrodes. Although a recent study demonstrated that PEDOT:PSS electrodes can deliver low-frequency electric fields without cytotoxicity [80], and another in vivo study showed that PEDOT:PSS coated electrodes could elicit responses in the visual cortex of mice for over a year [50], the longevity of our specific electrode design in vivo remains to be determined. A longitudinal study in mice, for instance periodically measuring electrode impedance with and without current application, would be necessary to assess long-term performance. Furthermore, potential heating of the electrode and tissue during stimulation requires further investigation. Experiments using thermal cameras and varying current levels will be necessary to characterize these thermal effects. In addition, histological analysis will be needed to assess potential tissue damage, including markers of inflammation, cell death, or gliosis [81].”*

• The abstract is very superficial in it’s current form with electric field value from simulation highlighted. More quantified data should form part of the abstract.

To address the reviewer's comment regarding the need for quantitative data in the abstract, we have incorporated the following sentences (line 28): *“We characterized the electrical and optical properties of the electrodes, focusing on the trade-off between the maximum current that can be delivered and optical transmittance. We found that a 1 mm diameter, 350 nm thick PEDOT:PSS electrode could be used to apply a maximum current of 130 μA while maintaining 84% transmittance (approximately 50% under 2-photon imaging conditions). We then evaluated the electrode performance in the brain of an anesthetized mouse by measuring the electric field with a nearby recording electrode and found values up to 30 V/m. Finally, we combined experimental data with a finite-element model of the in vivo experimental setup to estimate the distribution of the electric field underneath the electrode in the mouse brain.”*

• Page 5, Line 186: “PEDOT:PSS vs Ag/AgCl ([-0.6 0.8] V)” – I believe “[-0.6 0.8] V” is being used to discuss the potential window. I am of the opinion it should be replaced with explanatory representation to improve paper readability.

To improve readability and clarify the rationale for the tests, we have revised the sentence as follows (line 201): *“Finally, we applied test pulses (Figure 2E) to identify current values, in either the anodic or cathodic phases, that lead to voltages outside the water window, the range of potentials where water does not undergo significant electrolysis. Operating outside this range can cause undesirable electrochemical reactions, such as gas evolution and pH changes. For our PEDOT:PSS electrodes, the water window is -0.6 V to 0.8 V when using a Ag/AgCl reference electrode.”*
